# The Aberrant Activation of NLRP3 in Microsatellites Stability Colon Cancer Promotes M2 Macrophage Polarization Based on the TCGA Database and Tissue Microarray Analysis

**DOI:** 10.1002/cnr2.70470

**Published:** 2026-03-18

**Authors:** Li Lu, Menglin Wu, Xunzhen Jiang, Tong Liu, Weihua Fu, Weidong Li, Xue Li, Zhicheng Zhao

**Affiliations:** ^1^ Department of General Surgery Tianjin Medical University General Hospital Tianjin China; ^2^ Radiology Department Second Hospital of Tianjin Medical University Tianjin China

**Keywords:** colorectal cancer, immunotherapy, microsatellite stable (MSS), NLRP3 immunosome

## Abstract

**Background:**

Microsatellites stability (MSS) colon cancer patients exhibit a significant suppressive immune status, and the functional status of tumor NLRP3 immunosomes plays an important role in regulating the tumor immune microenvironment, but whether they are involved in the regulation of immunosuppression in MSS patients is unclear. Therefore, further exploration of the relevant molecular mechanisms is urgently needed.

**Methods:**

The Cancer Genome Atlas‐Colorectal Cancer (TCGA‐COAD) Masked Somatic Mutation data, clinicopathological data were obtained, analyzed, and visualized using the ‘maftools’ in R package. Tissue microarray (TMA) used for this study includes 100 unselected, non‐consecutive, primary, and sporadic CRCs treated between April 2006 and October 2010 in Tianjin Medical University General Hospital and 60 adjacent noncancerous tissues. Demographic and clinicopathological variables were collected, and the clinical value and prognostic impact of NLRP3 expression were analyzed. Tissue immunofluorescence (IF) was applied to investigate the colocalization expression of NLRP3 and ASC in tumor cells. The Vectra 3.0 Automated Quantitative Pathology Imaging System was used to obtain spectral information the NLRP3‐ASC colocalization was analyzed by the Fiji Plugin “Coloc2”. Cytotoxic T lymphocytes and M2 macrophages in tumor tissue were evaluated by immunohistochemistry.

**Results and Conclusion:**

In patients with MSS‐CRC, aberrant activation of NLRP3 immunosome was significantly associated with lymph node metastasis of tumors. It is also closely related to the polarization of M2 macrophages in the tumor microenvironment, and further affects the infiltration of CD8+T lymphocytes, thereby creating a suppressive immune microenvironment.

## 1 Introduction

Colorectal carcinoma (CRC) ranks as the third most prevalent malignancy globally across both genders, with epidemiological data documenting approximately 1.8 million incident cases and 880 000 cancer‐related deaths annually [1]. A pivotal molecular characteristic distinguishing CRC subtypes involves dysfunction in the DNA mismatch repair (MMR) mechanism [2]. This molecular defect drives microsatellite instability (MSI) in approximately 15% of CRC cases, contrasting with the 85% of tumors maintaining microsatellite stability (MSS) through intact MMR pathways [3]. Such molecular stratification underlies differential therapeutic responsiveness across CRC subtypes.

Current clinical management strategies encompass surgical resection, cytotoxic chemotherapy, and radiation therapy [4]. However, these conventional modalities frequently induce substantial off‐target effects due to their non‐selective action on rapidly proliferating cells [5]. This therapeutic limitation underscores the critical need for developing precision‐targeted interventions.

Emerging immunotherapeutic approaches represent a paradigm shift in oncology [6]. Unlike conventional therapies, these strategies harness host immune competence to selectively eradicate malignant cells through tumor antigen recognition [7]. The past decade has witnessed remarkable clinical breakthroughs, particularly in hematological malignancies and select solid tumors, where immunotherapy has demonstrated unprecedented durable responses [8, 9]. In CRC, checkpoint inhibitors gained regulatory approval in 2017, though their application remains restricted to hypermutated MSI‐high (MSI‐H) tumors [10]. The therapeutic efficacy in this subgroup correlates with elevated tumor mutational burden (TMB), whereas MSS tumors with low TMB (pMMR‐MSI‐L) exhibit inherent resistance [11].

Notably, TMB alone fails to fully account for immunotherapy resistance in MSS CRC, as evidenced by differential responses in non‐CRC malignancies with comparable mutation loads [12, 13]. Intriguingly, a MSS subpopulation displays MSI‐H‐like immunophenotypes characterized by immune cell infiltration and neoantigen expression, correlating with improved clinical outcomes [14, 15]. These observations suggest alternative mechanisms beyond TMB may govern immunotherapy responsiveness.

For MSS CRC, reduced lymphocyte infiltration and the presence of “cold tumors” are common characteristics [16]. Therefore, elucidating the mechanisms by which MSS CRC creates an inhibitory immune microenvironment is of critical importance. Recently, growing evidence has indicated that tumor cells can modulate the functions of immune cells within the tumor microenvironment, particularly macrophages and tumor‐killing effector T lymphocytes, by secreting specific immune regulatory factors [17, 18]. Studies on inflammation‐associated colorectal cancer have shown that tumor cells' own inflammation‐related factors can regulate the function of immune cells in the tumor microenvironment, thereby modulating the tumor immune environment and ultimately inducing tumorigenesis [19, 20, 21]. Among these, the NLRP3 inflammasome plays a significant role [22, 23]. Therefore, the NLRP3 inflammasome may be involved in regulating the immune microenvironment in MSS colorectal cancer patients; however, research on its mechanisms remains limited.

The NLRP3 inflammasome, a multiprotein complex within the Nod‐like receptor family, comprises three core components: NLRP3 scaffold proteins, PYCARD/ASC adaptors, and caspase‐1 effectors [24]. This molecular sensor responds to diverse pathogen‐ and damage‐associated molecular patterns (PAMPs/DAMPs), with dysregulation implicated in various inflammatory disorders [25, 26]. This protein complex consists of three components including (a) NLRP3 scaffold, (b) PYCARD (PYD And CARD Domain) adaptor, frequently referred to as apoptosis‐associated speck‐like protein (ASC), which functions as a caspase‐1 activator, and (c) the third component, which is caspase‐1 [27]. The role of the NLRP3 inflammasome in human cancers remains a subject of considerable debate [28, 29]. Substantial evidence indicates that NLRP3 inflammasome activation is linked to tumor advancement, patient prognosis, and responses to therapy [30]. On the other hand, several studies have highlighted its potential anti‐tumor functions. For instance, Ghiringhelli [31] demonstrated that the NLRP3 inflammasome is essential for dendritic cells to prime T lymphocytes capable of producing IFN‐γ in response to tumor cells. Additionally, deficiency in NLRP3 inflammasome signaling has been associated with elevated tumor burden in colorectal cancer models [32]. Dupaul‐Chicoine [33] further reported that NLRP3‐dependent production of IL‐18 restrains metastatic growth of colorectal cancer in the liver. Another study supports that the NLRP3 inflammasome suppresses hepatic metastasis of colorectal cancer by enhancing the tumor‐killing activity of natural killer (NK) cells via an IL‐18‐mediated mechanism that operates independently of IFN‐γ; this conclusion is reinforced by observations of increased liver metastases in NLRP3 inflammasome knockout mice. Furthermore, Mutala [34] suggested that inflammasome stimulation promotes the recruitment of tumor‐infiltrating lymphocytes (TILs) through the Caspase‐1/IL‐18 axis.

Accumulating evidence indicates that NLRP3 expression in tumor tissues is widely distributed across multiple cell types. It can be derived from immune cells within the tumor microenvironment as well as from tumor cells themselves. The mechanisms through which NLRP3 exerts its functions vary depending on the cellular source and context, potentially leading to divergent functional outcomes. This heterogeneity may partly account for the ongoing controversies regarding its role in tumor immunity. Therefore, clarifying the expression patterns of NLRP3 in colorectal cancer and its impact on the immune microenvironment is critical for elucidating the functional crosstalk between tumor cells and immune cells in MSS colorectal cancer, thereby providing deeper mechanistic insights into the immunoregulatory processes in this tumor subtype.

In the present study, we analyzed public data sets and used 298 cases from The Cancer Genome Atlas (TCGA), with a focus on the mRNAseq data difference between primary NLRP3 and activation NLRP3; we also explored the ability of the NLRP3 inflammasome to predict tumor immunity compared with the signatures that have been reported. Based on the above analysis, we utilized a tissue microarray (TMA) approach including 100 well‐documented, clinically annotated colorectal cancer specimens to investigate the colocalization expression of NLRP3 and ASC in tumor cells by tissue immunofluorescence (IF), which can imply the NLRP3 inflammasome status precisely and concomitantly focusing on its association with the clinicopathological and prognostic value. The TMA is founded on the rationale that each tissue spot can be taken as a model of an immune microenvironment. We counted CD8^+^ T cells and CD206^+^ M2 macrophages for each tissue spot, as defined by immunohistochemistry. The correlation between NLRP3 inflammasome status and immune cells infiltrating was also assessed ultimately. Our approach implicated the association of NLRP3 inflammasome activation and antitumor immunity in MSS CRC.

## 2 Materials and Methods

### 2.1 Data Acquisition and Processing

Somatic mutation profiles from TCGA‐COAD (varscan.somatic.maf) were processed and downloaded from the TCGA database (https://portal.gdc.cancer.gov/analysis_page?app=Downloads) and visualized through the R package ‘maftools’ [35]. Gene expression data (RNAseqV2) and clinical records were retrieved from the TCGA portal (https://portal.gdc.cancer.gov/) as of August 1, 2021. Our cohort comprised 298 CRC cases with normalized transcriptomic profiles and complete clinical annotations. Expression values underwent log2 transformation of FPKM metrics using the ‘limma’ package (R v3.6.0). |log_2_FC| > 1 and a *p* < 0.05 were applied for identifying differentially expressed genes. Microsatellite‐stable (MSS) cases were defined by wildtype status across mismatch repair genes, with exclusion criteria applied to specimens lacking pretreatment records, incomplete survival data, or fragmented RNAseq information. Clinicopathological variables including demographics, tumor localization, histology, and TNM staging were systematically cataloged.

### 2.2 Inflammasome Activity Quantification

The NLRP3 inflammasome score was calculated based on the single‐sample gene‐set enrichment analysis (ssGSEA) using the NLRP3‐inflammasome‐related gene set to quantify the expression levels of these genes for MSS‐CRC from the TCGA [36]. Median value of the NLRP3 inflammasome score was defined as the cut‐off value between the high NLRP3 group and the low NLRP3 group.

### 2.3 Immune Microenvironment Profiling

The CIBERSORT algorithm [37] (R implementation) deconvoluted tumor‐infiltrating immune cell (TIIC) proportions from RNAseq data. Quantile normalization was disabled per developer recommendations. Twenty‐two immune subsets were quantified: naïve B cells (Bn), memory B cells (Bm), plasma cells, CD8+ T cells, CD4+ T cell subsets (naïve [Tn], resting memory [Tmr], activated memory [Tma]), follicular helper T cells (Tfh), regulatory T cells (Tregs), γδ T cells, natural killer subsets (resting [NKr], activated [NKa]), monocytes, macrophage polarization states (M0/M1/M2), dendritic cell subsets (resting [DCr], activated [DCa]), mast cell subtypes (resting [Mr], activated [Ma]), eosinophils, and neutrophils.

### 2.4 Cohort Characteristics and TMA Design

A tissue microarray (TMA) incorporating 100 primary CRC specimens (2006–2010, Tianjin Medical University General Hospital) and 60 adjacent normal tissues was constructed as previously validated [38]. Core sampling (0.6 mm diameter) ensured ≥ 50% tumor cellularity per spot. Clinicopathological parameters encompassed age, sex, tumor site, histopathology, TNM staging, differentiation grade, vascular/neural invasion, lymph node yield. MMR‐proficient tumors were defined as those simultaneously expressing MutL homolog 1 (MLH1), MutS homolog 2 (MSH2), MutS homolog 6 (MSH6) and postmeiotic segregation increased 2 (PMS2), while MMR‐deficient tumors were defined as those lacking expression of at least one of these markers. Ethical compliance was confirmed through institutional review board approval (IRB2020 KY 640) with informed consent obtained per Declaration of Helsinki guidelines. Approval was granted by the Ethics Committee of Tianjin Medical University General Hospital.

### 2.5 Immunohistochemical Staining of MMR Genes

Tissue sections were deparaffinized and subjected to antigen retrieval using Ventana CC1 buffer. Primary antibodies against human DNA mismatch repair proteins—including mouse anti‐MLH1 (clone G168‐15, catalog #ab14206; Abcam), mouse anti‐MSH2 (clone 44, catalog #ab52266; Abcam), mouse anti‐MSH6 (clone G168‐15, catalog #ab14204; Abcam), and rabbit anti‐PMS2 (clone EPR3947, catalog #ab214442; Abcam)—were diluted at a ratio of 1:300. A volume of 50 μL of the diluted antibody mixture was applied to the colorectal cancer tissue sections and incubated at 4°C for 24 h. Afterward, the slides were allowed to equilibrate at 37°C for 30 min. Subsequently, a secondary antibody was applied, followed by incubation at 37°C for another 30 min. Detection was performed using horseradish peroxidase (HRP) and 3,3′‐diaminobenzidine (DAB) chromogen for 30 min. Finally, the slides were mounted with resin. For result interpretation, two pathologists independently evaluated the staining under double‐blind conditions. Multiple fields of view per section were examined under 400× magnification. Brownish‐yellow staining was considered positive. Samples were classified as negative if fewer than 30% of the cells showed positive staining, and positive if 30% or more of the cells were stained.

### 2.6 Multiplex Immunofluorescence Protocol

Spatial co‐expression of NLRP3 and ASC was assessed via Opal 7‐Color IHC (PerkinElmer) on 4 μm TMA sections [39]. Sequential staining involved: (1) Antigen retrieval (citrate buffer, pH 6.0), (2) primary antibody incubation (CK‐AE1/AE3, NLRP3‐77080, ASC‐61682), (3) Opal polymer‐HRP conjugation, (4) tyramide signal amplification (TSA) with spectral fluorophores (CK‐Opal690, NLRP3‐Opal520, ASC‐Opal570). DAPI counterstaining facilitated nuclear localization. Technical validation followed established protocols [40].

### 2.7 Quantitative Imaging Workflow

Vectra 3.0 automated microscopy (PerkinElmer) acquired multispectral images at 20× magnification [41]. Inform 2.2 software performed spectral unmixing and colocalization analysis via Fiji's Coloc2 plugin, with Pearson's *R* ≥ 0.5 defining NLRP3‐ASC positive clusters [42].

### 2.8 Immune Cell Quantification

CD8+ cytotoxic T lymphocytes and CD206+ M2 macrophages were enumerated using ImageJ‐based analysis: (1) Color deconvolution (DAB channel thresholding), (2) pixel density calculation (positive cells/mm^2^), (3) manual cell counting with ocular grid calibration. Stromal and intraepithelial compartments were analyzed separately.

### 2.9 Statistical Analysis

The data were analyzed with the Mann–Whitney U test or chi‐square test for expressing the differences in immune cellular densities, and overall survival was investigated using the Kaplan–Meier analysis so that we were able to find significant prognostic markers. SPSS 22.0 software (SPSS, Chicago, IL, USA) and GraphPad Prism 5.0 software (GraphPad Software, La Jolla, CA, USA) were used for statistical analyses. All *p*‐values < 0.05 were considered to be statistically significant.

## 3 Results

1.TCGA database data analysis

(1). TCGA mutation database data download

In this study, we downloaded mutation data from TCGA database of colon cancer patients, totaling 399 patients. By analyzing the mutations of MSH6, MSH2, MLH1, PMS2 mismatch repair genes, the patients were divided into MSS group and MSI group, in which 354 patients were MSS and 45 patients were MSI group. The detailed information of patients categorized by MMR groups and the gene mutation data for the MSI group are provided in Data S1 and S2, respectively.

(2) TCGA transcriptome data download

The transcriptome data of colon cancer patients in TCGA database were downloaded for a total of 380 patients, and the intersection of this patients with 354 MSS patients in the mutation database was taken to obtain a total of 296 patients. NLRP3 expression was classified into high and low expression groups using the NLRP3 ES score, including 120 cases in the NLRP3 high expression group and 176 cases in the low expression group.

(3) Profiling tumor‐infiltrating immune cells (CIBERSORT) lymphocyte infiltration analysis.

**FIGURE 1:**
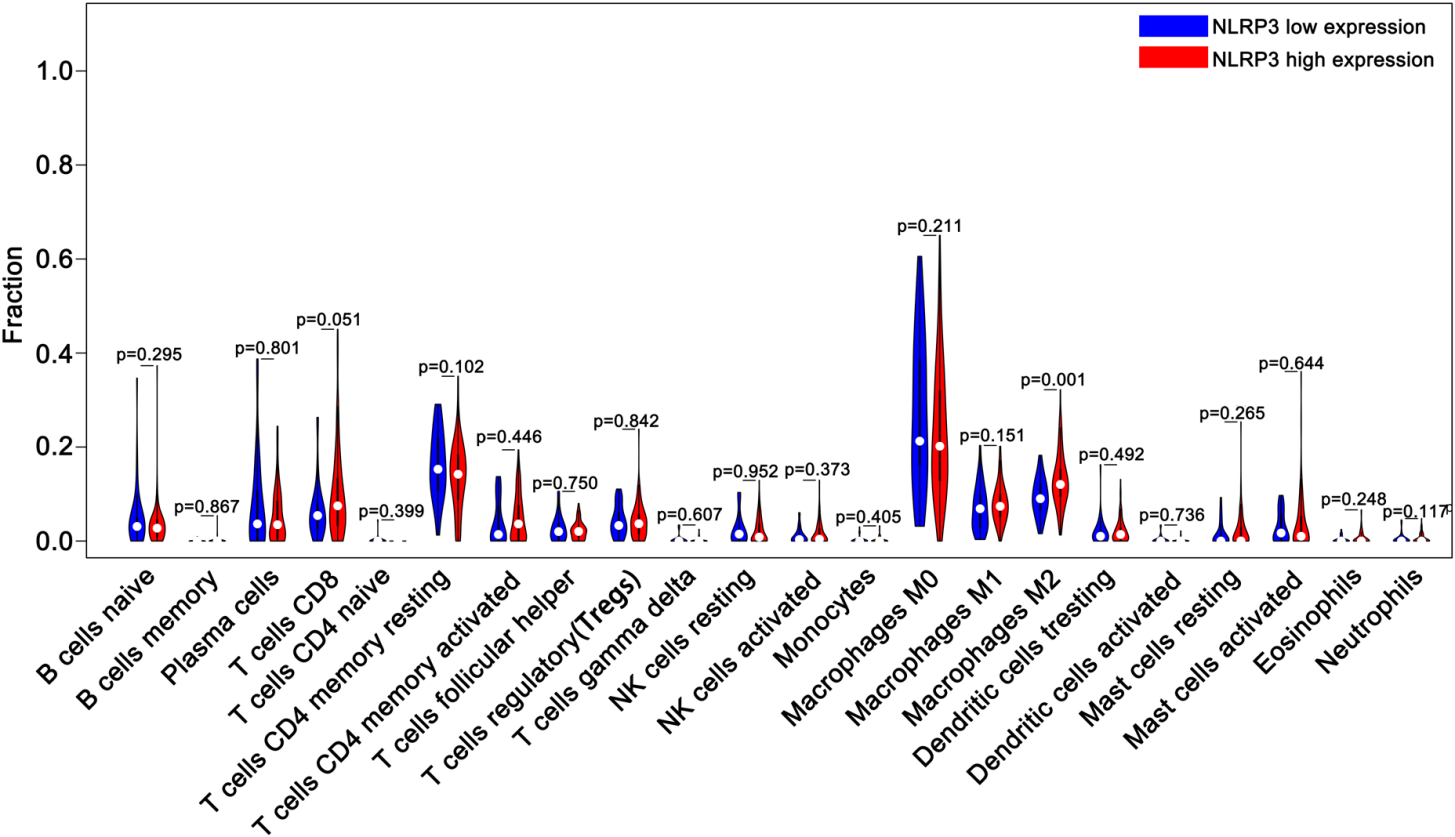
Comparison of lymphocyte infiltration at different expression levels of NLRP3 by CIBERSORT.

Based on the above NLRP3 grouping results, this study compared CRC tumor‐infiltrating lymphocytes in different NLRP3 groupings by analyzing transcriptome data and using CIBERSORT. The results showed that the infiltration level of M2 macrophages in the NLRP3 high‐expression group was significantly higher than that in the NLRP3 low‐expression group (*p* = 0.001), and the level of CD8 T‐lymphocyte infiltration, which was also higher in the NLRP3 high‐expression group than in the NLRP3 low‐expression group, was not statistically(*p* = 0.051) (Figure 1).

(4) Clinicopathological factors and survival analysis of NLRP3 different expression groups in MSS patients.

The clinical data of colon cancer patients in the TCGA database were downloaded for a total of 385 patients, and the patients were intersected with 298 MSS patients in the mutation database to obtain a total of 284 patients with complete clinical data and follow‐up data, including 112 patients in the NLRP3 high expression group and 172 patients in the low expression group. By analyzing the clinicopathological data of MSS‐CRC patients in the TCGA database, the results showed that patients in the NLRP3 high‐expression group were more likely to develop lymph node metastasis, and the N stage was higher than that of the NLRP3 low‐expression group, and the difference was statistically significant (*p* = 0.015). No statistical difference was found between the two groups in T‐stage, M‐stage and AJCC pathologic staging (Table 1).

**TABLE 1:** Comparison of clinicopathological factors for MSS CRC patients between different expression levels of NLRP3.

Clinicopathological factors	NLRP3‐high	NLRP3‐low	*X* ^2^ *	*P*
Gender			1.251	0.263
male	61	82		
female	51	90		
Year			1.342	0.247
≤ 65	43	78		
> 65	69	94		
T stage			0.551	0.458
T1	4	4		
T2	21	30		
T3	76	118		
T4	11	20		
N stage			5.905	**0.015**
N0	55	106		
N1	28	40		
N2	29	26		
M stage			0.123	0.726
M0	98	148		
M1	14	24		
AJCC			0.803	0.370
I	21	31		
II	34	66		
III	37	51		
IV	20	24		

* Mann–Whitney U test or chi‐square test was applied in this table.

**FIGURE 2:**
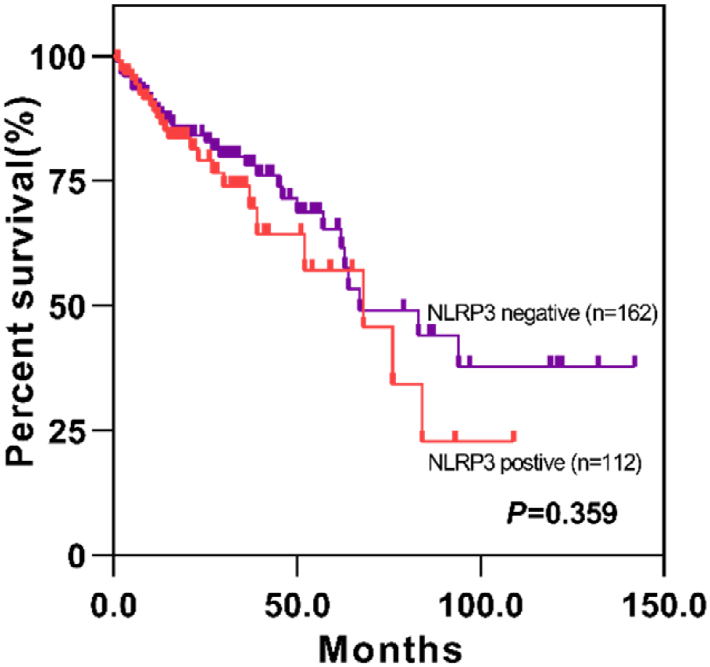
Survival analysis of MSS CRC patients at different expression levels of NLRP3.

We collected clinical data from patients in the TCGA database, survival data of 162 patients in NLRP3‐negative group and 112 in the NLRP3‐positive group were available. Based on log‐rank tests across NLRP3 expression, the negative group was associated with a favorable prognosis, but no statistically significant difference compared with positive NLRP3 expression group. (*p* = 0.359) (Figure 2).

2. Colon cancer tissue microarray analysis

(1). Tissue microarray MMR expression detection

**FIGURE 3:**
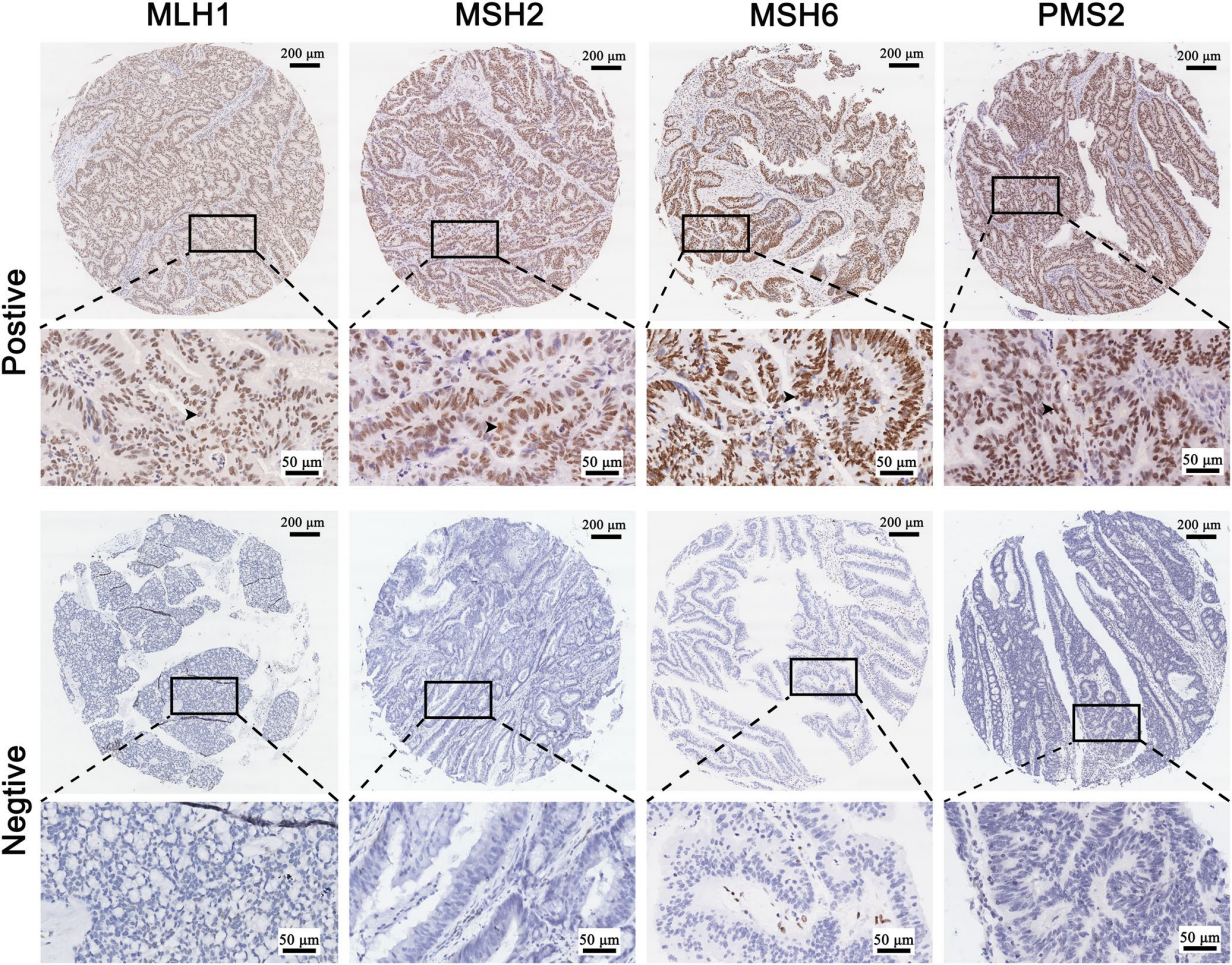
Immunohistochemical detection of MMR gene expression level of TMA. (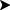 **:** Expression location of MMR protein).

The IHC of MSH6, MSH2, MLH1, and PMS2 mismatch repair genes in tissue microarrays were detected in 100 patients excluding 4 cases with poor staining quality, totaling 96 cases. Based on the expression of MMR, patients were categorized into MSS and MSI groups. Based on previous studies, Patients with positive expression of all four genes (MSH6, MSH2, MLH1, and PMS2) were categorized into the MSS group, whereas those with negative expression of one or more genes were assigned to the MSI group. Ultimately, our cohort comprised 32 patients in the MSI group and 64 patients in the MSS group. (Figure 3).

(2) Colorectal cancer tissue microarray NLRP3‐ASC co‐localization staining analysis.

**FIGURE 4:**
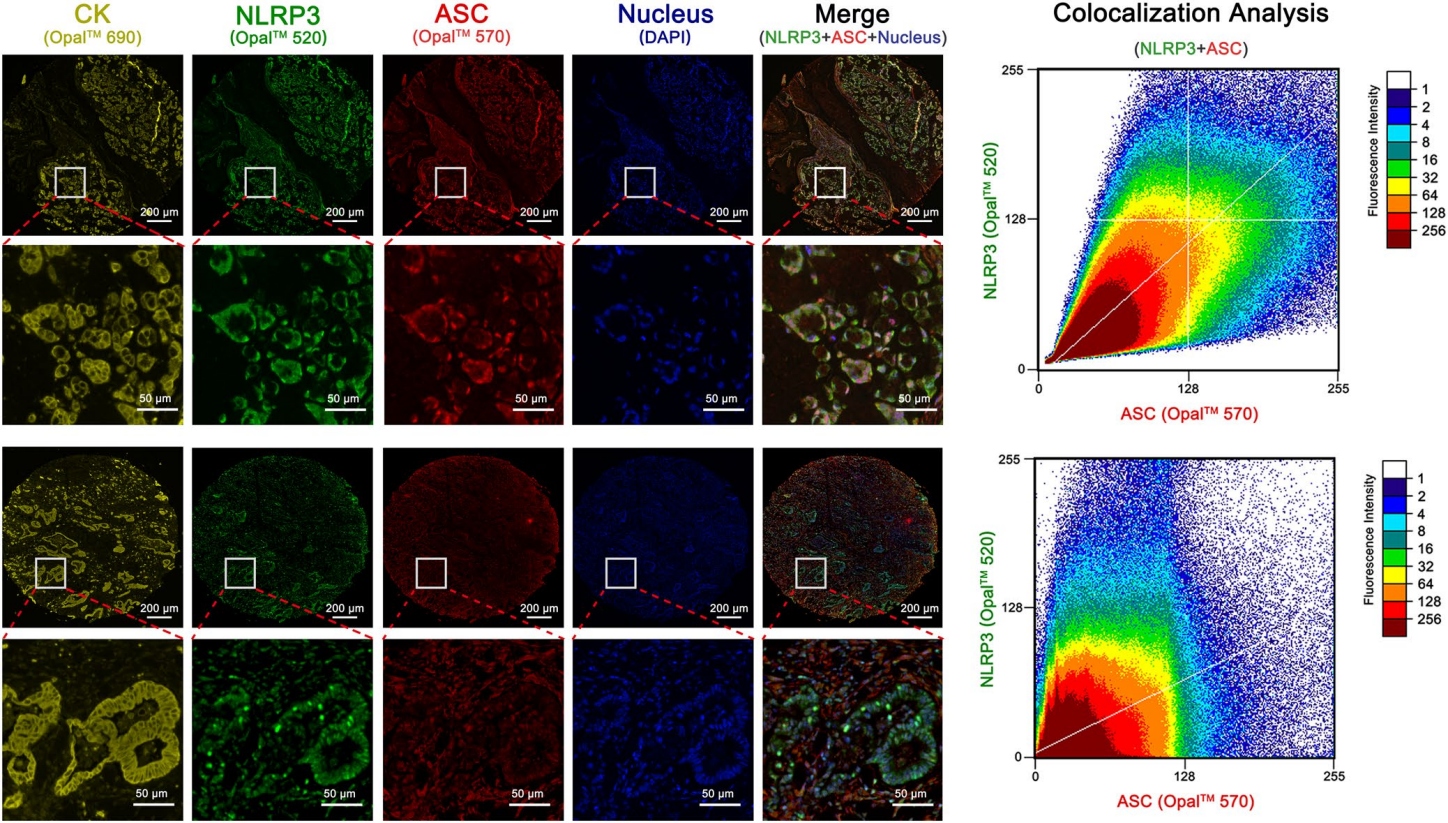
Multicolor immunofluorescence co‐localization detection of ASC‐NLRP3 immunosome activation of TMA.

The expression of NLRP3‐ASC was detected using a dual fluorescence co‐localization method, and patients were divided into NLRP3 activation group and NLRP3 silencing group according to the median grouping of co‐localization indices. Of the 64 MSS patients included, 30 cases were in the NLRP3 positive group, and 34 cases were in the NLRP3 negative group (Figure 4).

(3) Effect of NLRP3 activation on clinicopathological factors and survival of MSS patients.

By analyzing the clinicopathological data of patients with tissue microarrays, it was shown that patients with colon cancer with NLRP3 activating were more likely to develop lymph node metastasis, and the difference was statistically significant compared with the group with NLRP3 silencing (*p* = 0.002). Meanwhile, the difference between the two groups of AJCC staging was statistically significant(*p* = 0.006). However, there was no statistically significant difference between T‐stage and M‐stage (Table 2).

**TABLE 2:** Comparison of clinicopathological factors for MSS CRC patients between different expression levels of NLRP3.

Clinicopathological factors	NLRP3‐high	NLRP3‐low	*X* ^2^ *	*P*
Gender			0.195	0.659
male	16	20		
female	14	14		
Year			0.042	0.832
≤ 65	16	19		
> 65	14	15		
Tumor location			0.195	0.659
Right colon	14	14		
Left colon	16	20		
Pathological type			2.121	0.346
Infiltrating type	3	6		
ulcerative type	22	19		
bulging type	5	9		
T stage			1.988	0.159
T1	0	0		
T2	1	2		
T3	6	12		
T4	23	20		
N stage			9.203	**0.002**
N0	9	23		
N1	12	8		
N2	9	3		
M stage			1.151	0.283
M0	29	34		
M1	1	0		
AJCC			7.524	**0.006**
I	1	1		
II	8	22		
III	20	11		
IV	1	0		

* Mann–Whitney U test or chi‐square test was applied in this table.

(4) Survival analysis.

**FIGURE 5:**
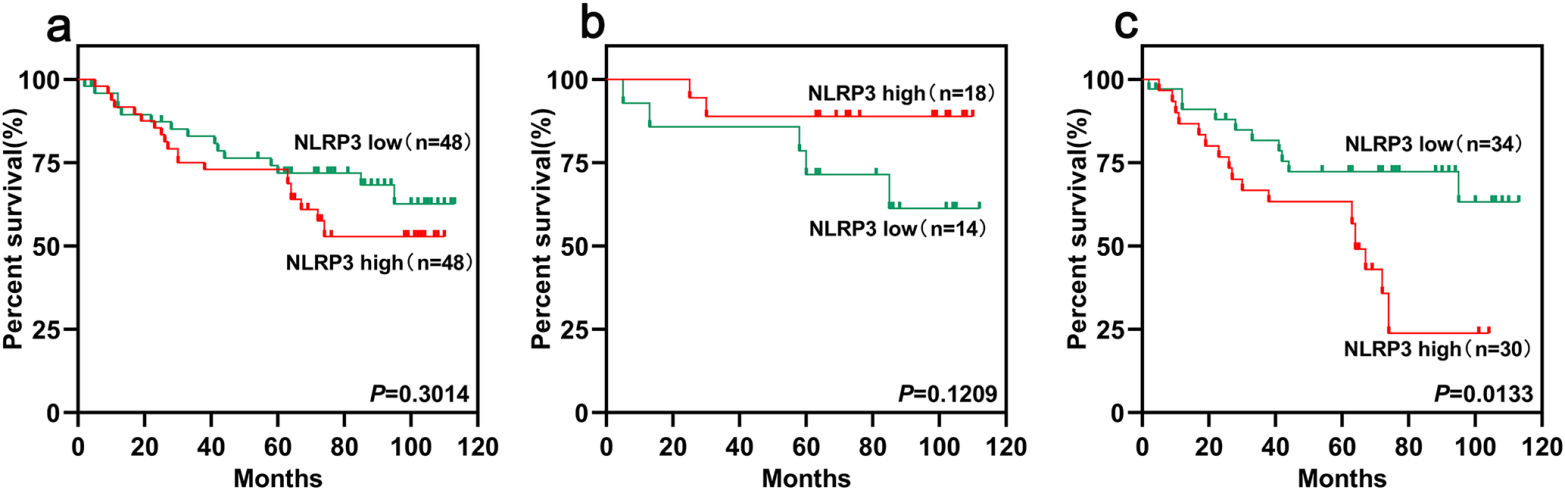
Survival analysis of CRC patients at different expression levels of NLRP3. (a) survival analysis of MSS and MSI CRC patients at different expression levels of NLRP3; (b) survival analysis at different expression levels of NLRP3 for MSI patients; (c) survival analysis at different expression levels of NLRP3 for MSS patients.

For all colon cancer patients, the difference of overall survival between activating and silencing NLRP3 groups was not statistically significant (*p* = 0.3014), and for MSI patients, no significant difference was observed (*p* = 0.1209), and for MSS patients, the prognosis of NLRP3‐activated patients was significantly worse than NLRP3‐silenced patients, and the difference between the two was statistically significant (*p* = 0.0133) (Figure 5).

3. Impact of CRC CD8/CD206 lymphocyte infiltration on clinicopathological factors and prognosis.

(1) Immunohistochemical staining assessment.

**FIGURE 6:**
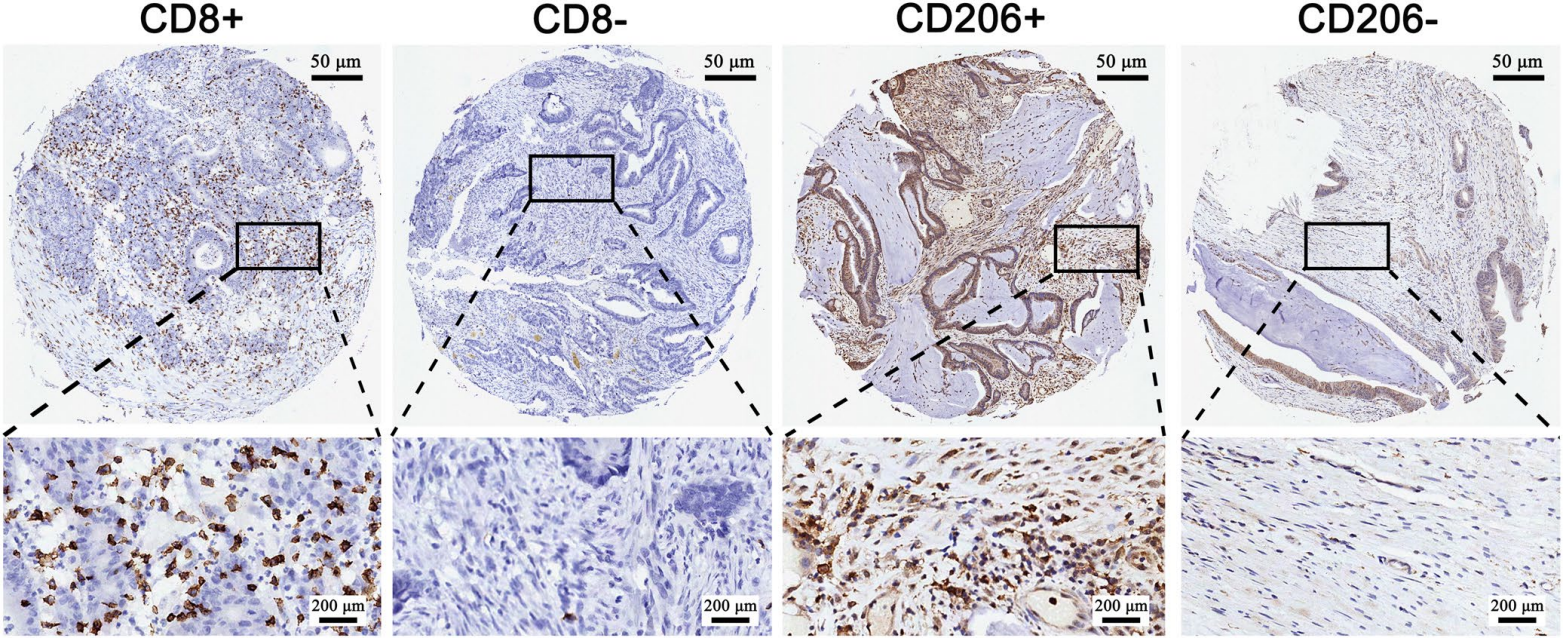
Immunohistochemical detection of CD8 and CD206 expression level of TMA.

There were 17 patients with high CD8 expression and 79 patients with low expression; 68 patients with high CD206 expression and 28 patients with low expression; 12 patients with high CD8 expression and 52 patients with low expression and 41 patients with high CD206 expression and 23 patients with low expression in MSS (Figure 6).

(2) Effect of CRC CD8/CD206 lymphocyte infiltration on clinicopathological factors in MSS patients.

We also performed immunohistochemistry for CD8 and CD206 expression in the tissue microarrays and divided the patients into CD8‐positive and CD8‐negative groups, and CD206‐positive and CD206‐negative groups according to the median expression index. Among them, 12 patients were CD8+ 52 patients were CD8‐; 41 patients were CD206+ and 23 patients were CD206‐. By comparing the differences in their clinicopathological factors, the results showed that CD8 lymphocyte infiltration was correlated with lymph node metastasis of tumors (*p* = 0.01), and correlated with the pathological stage of AJCC (*p* = 0.039); M2 macrophage polarization was similarly correlated with lymph node metastasis of CRC, and the tumors with high infiltration of M2 macrophage were more likely to have lymph node metastasis (*p* = 0.022); and for the two groups in the Comparison of NLRP3 expression showed that patients in the NLRP3+ group had significantly less CD8 lymphocyte infiltration (*p* = 0.02) and significantly more M2 macrophage infiltration (*p* = 0.047) (Table 3).

**TABLE 3:** Comparison of clinicopathological factors for MSS CRC patients between different expression levels of CD8 and CD206.

Clinicopathological factors	CD8+	CD8‐	*X* ^2^	*P*	CD206+	CD206‐	*X* ^2^ *	*P*
Gender			0.651	0.420			0.311	0.577
male	8	28			22	14		
female	4	24			19	9		
Year			1.010	0.315			0.554	0.457
≤ 65	5	30			21	14		
> 65	7	22			20	9		
Tumor location			0.026	0.872			1.173	0.279
Right colon	5	23			20	8		
Left colon	7	29			21	15		
Pathological type			3.445	0.179			0.893	0.640
infiltrating type	1	8			5	4		
ulcerative type	6	35			28	13		
bulging type	5	9			8	6		
T stage			0.692	0.405			0.385	0.535
T1	0	0			0	0		
T2	1	2			2	1		
T3	4	14			10	8		
T4	7	36			29	14		
N stage			6.686	**0.010**			5.257	**0.022**
N0	10	22			17	15		
N1	2	18			13	7		
N2	0	12			11	1		
M stage			0.231	0.631			1.783	0.182
M0	12	51			41	22		
M1	0	1			0	1		
AJCC			**4.272**	**0.039**			0.891	0.345
I	0	2			2	0		
II	10	20			15	15		
III	2	29			24	7		
IV	0	1			0	1		
NLRP3			5.412	**0.020**			3.897	**0.047**
+	2	28			23	7		
—	10	24			18	16		

* Mann–Whitney U test or chi‐square test was applied in this table.

(3) Effect of CD8/CD206 lymphocyte infiltration on the prognosis of CRC patients.

**FIGURE 7:**
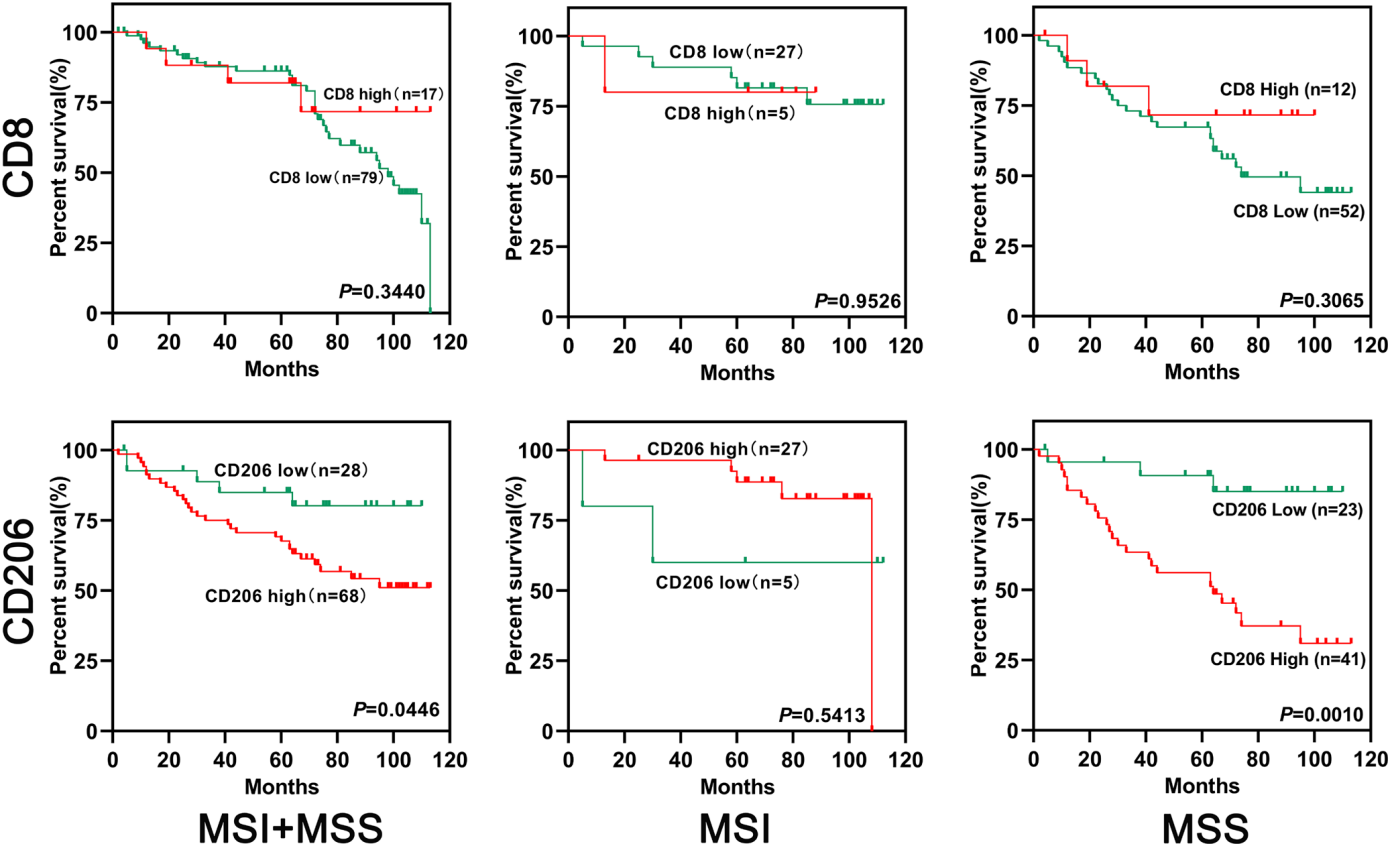
Survival analysis of CRC patients at different expression levels of CD8 and CD206.

Based on the data of this study, for overall colon cancer patients, there was no statistical difference in survival between the high and low groups of CD8+ lymphocyte infiltration of tumor tissue (*p* = 0.3440), for MSI patients, the same results could be obtained (*p* = 0.9526), and for MSS patients, the prognosis of patients with high CD8 expression was better than that of patients with low CD8 expression, but there was no statistical difference between the two (*p* = 0.3065). For overall colon cancer patients, the prognosis of patients in the group with high CD206 expression in tumor tissue was worse and significantly different from those in the low expression group (*p* = 0.0446); for MSI patients, there was no statistical difference between the two groups (*p* = 0.5413), and for MSS patients, the prognosis of patients with high CD206 expression was worse and significantly different from those in the low expression group (*p* = 0.0010) (Figure 7).

## 4 Discussion

The prognosis for colorectal cancer patients is still not favorable despite efforts to understand the causes and develop better screening and treatment methods. Recent research on molecular typing of colorectal cancer has revealed that the functional status of MMR, the mutational load of the tumor, and the status of microsatellites have a significant impact on patients' prognosis. Patients with dMMR or MSI‐H tend to have higher mutational loads, and their tumors are heavily infiltrated with lymphocytes in the tumor microenvironment, leading to worse tumor invasion and metastasis. These patients tend to respond well to immunotherapy. As a result, detecting the MMR gene has become an important consideration for postoperative adjuvant treatment of colon cancer. However, it has been gradually found that only a small number of colon cancer patients have MSI‐H status; especially for sporadic colon cancer, most of the patients have MSS status, and this part of the patients have a strong invasive and metastatic ability of the tumor cells, with little lymphocyte infiltration in the microenvironment, and poor response to immunotherapy, which is referred to as immune escape, and the overall prognosis of this part of the patients is significantly poorer than that of MSI‐H patients. The overall prognosis of this group of colon cancer patients was significantly worse than that of MSI‐H patients. Therefore, clarifying the mechanism of immune escape in MSS patients is of great significance for improving the prognosis of MSS colon cancer patients, enhancing the response to immunotherapy, and further developing novel therapeutic targets for MSS patients. In this study, we initially utilized the TCGA database to examine colon cancer‐related data. We aimed to analyze the mutation database and identify MSS patients with mutations in MSH2, MLH1, PMS2, and MSH6. This approach was consistent with the method employed in Lin's study [43].

The NLRP3 inflammasome constitutes a multimolecular assembly comprising three core elements: an apoptosis‐associated speck‐like protein containing a caspase recruitment domain (ASC), the NLRP3 scaffold, and procaspase‐1 precursors. Upon activation, this complex facilitates ASC‐mediated proteolytic cleavage of procaspase‐1 into its active form, initiating inflammatory cascades through IL‐1β and IL‐18 maturation. Mounting evidence implicates NLRP3 signaling in oncogenesis across multiple malignancies, including colitis‐associated carcinomas, pulmonary neoplasms, mammary tumors, and cutaneous melanomas [43, 44]. In colorectal carcinogenesis, murine models demonstrate contrasting phenotypic outcomes: NLRP3‐deficient and IL‐18‐knockout strains exhibit heightened susceptibility to dextran sulfate sodium‐induced colitis and tumorigenesis [22], whereas inflammasome activation exerts protective effects against colitis‐associated dysplasia [45]. Discrepancies in reported tumorigenic roles may stem from differential NLRP3 expression patterns between stromal and epithelial compartments [46, 47]. Bulk transcriptomic analyses inherently obscure cellular localization, potentially confounding tissue‐level expression interpretations. To address this limitation, we implemented single‐sample gene set enrichment analysis (ssGSEA) using a curated NLRP3 inflammasome gene signature [48], mirroring methodologies established in prior computational immunology studies [36]. This computational framework enables precise quantification of functional inflammasome activity within malignant cells, circumventing spatial resolution constraints of conventional sequencing approaches. Based on the aforementioned grouping criteria, we first compared the extent of lymphocyte infiltration in tumor tissues between the two patient groups. For this analysis, we employed the “CIBERSORT” algorithm, a widely utilized method for immune cell enumeration that has been extensively validated in numerous previous studies [49, 50]. Our findings revealed that patients in the high NLRP3 expression group exhibited a notable increase in M2 macrophages and a reduction in T lymphocyte infiltration within the tumor microenvironment, although these differences did not reach statistical significance. Consistent with our observations, a study by Chen [51] demonstrated that activation of the NLRP3 inflammasome promotes M2 macrophage polarization, thereby modulating the immune microenvironment—a conclusion that aligns with the trends identified in our cohort. Furthermore, research by Wu [52] indicated that NLRP3 inflammasome activation facilitates tumor cell invasion and metastasis by inducing epithelial–mesenchymal transition (EMT). This mechanism may explain why patients with high NLRP3 expression are more prone to lymph node metastasis, a pattern also supported by our analysis of clinicopathological factors using tissue microarrays.

In this research, we assessed the clinical significance of NLRP3 expression by using tissue microarrays. We conducted immunohistochemistry to identify MMR gene and found that patients with positive MLH1, MSH2, MSH6, PMS2 were classified as MSS patients, consistent with the method used in Chen's study [53]. Moreover, we differentiated the expression of NLRP3 from tumor cells and mesenchymal origin using CK expression, which allowed for accurate spatial localization of tissue microarray protein expression, a method similar to the one used in Rieger G's study [54]. This approach compensated for the drawback of the inaccurate localization of expression in high‐throughput sequencing results. Previous studies have shown [55, 56], that the functional form of NLRP3 immunosome is NLRP3‐ASC binding, and this binding state is taken as the functional form of the immunosome. Therefore, the high and low expression of NLRP3 was not used as a criterion for the high and low function of the immunosome in the present study, but rather, immunofluorescence co‐localization was used to determine the method of NLRP3 activation. In the analysis of TCGA data, this study employed an NLRP3 scoring system to categorize NLRP3 expression levels. It is important to note that this approach differs from the method used in our TMA analysis, in which multiplex immunofluorescence was applied to determine NLRP3 expression status. The latter method is considered more accurate and objective, as it allows for precise spatial and quantitative assessment of protein expression within tissue specimens. Therefore, we propose that the discrepancy in methodology for group stratification may contribute to the differences observed in survival analyses between the online database cohort and our institution's patient cohort. The analysis of clinicopathological factors revealed that in MSS CRC, high expression of NLRP3 was linked to lymph node metastasis, consistent with the study's database and a previous study [57]. When assessing the prognostic value of NLRP3, it was observed that in the intact CRC patient group and MSI‐H patients, the expression of NLRP3 did not significantly correlate with patient prognosis. However, in MSS patients, those with high NLRP3 expression had a worse prognosis. In our analysis of lymphocyte infiltration and patient prognosis, we observed that the extent of CD206^+^ M2 macrophage infiltration was negatively associated with prognosis, which is consistent with the findings reported by Väyrynen [58]. However, although high infiltration of CD8^+^ T lymphocytes showed a trend toward better prognosis, the difference compared to the low infiltration group did not reach statistical significance. This contrasts with certain previous studies. We speculate that this discrepancy may be influenced by the limited sample size—specifically, only 12 MSS CRC patients in this study exhibited high CD8^+^ T lymphocyte infiltration, which may have affected the statistical power of the survival analysis. We plan to further expand the cohort and refine the survival analysis in subsequent studies.

So far, as far as our findings are concerned, this study is the first to explore the link between NLRP3 expression and immune escape in MSS colon cancer. This provides a foundation for further investigation into the immune escape mechanism of MSS colon cancer and the identification of new therapeutic targets. Nevertheless, our study has several limitations that should be addressed in future work. First, this research was primarily focused on the correlation between NLRP3 expression levels and M2 macrophage polarization in the tumor microenvironment, thus representing a purely correlational study. It lacks sufficient evidence for causal inference and mechanistic insight. Based on the findings presented here, our subsequent experiments will further investigate the mechanism by which NLRP3 induces M2 macrophage polarization in the tumor microenvironment.

## Author Contributions

**Li Lu:** project administration.

## Funding

This research was funded by Tianjin Health Research Project, TJWJ2021QN035 and National Natural Science Foundation of China, 82003301.

## Conflicts of Interest

The authors declare no conflicts of interest.

## Supporting information


**Data S1:** MMR Patient Grouping. The stratification of patients into microsatellite stable (MSS) and microsatellite unstable (MSI) groups, according to their MMR gene mutation profiles, is presented, with details of patient IDs provided in the table.

**Data S2:** Mutation Data. This table lists the detailed mutational profiles of 45 patients identified with MMR gene mutations. The data were obtained by downloading and screening the colorectal cancer mutation dataset from The Cancer Genome Atlas (TCGA).

## Data Availability

The data that support the findings of this study are openly available in TCGA at https://portal.gdc.cancer.gov/.
